# Overexpression of *Hevea brasiliensis HbICE1* Enhances Cold Tolerance in *Arabidopsis*

**DOI:** 10.3389/fpls.2017.01462

**Published:** 2017-08-22

**Authors:** Hong-Mei Yuan, Ying Sheng, Wei-Jie Chen, Yu-Qing Lu, Xiao Tang, Mo Ou-Yang, Xi Huang

**Affiliations:** ^1^Hainan Key Laboratory for Sustainable Utilization of Tropical Bioresources, Institute of Tropical Agriculture and Forestry, Hainan University Haikou, China; ^2^State Key Laboratory of Hybrid Rice, College of Life Sciences, Wuhan University Wuhan, China

**Keywords:** *hevea brasiliensis*, *ICE1*, cold stress, CBF pathway, bHLH, reactive oxygen species

## Abstract

Rubber trees (*Hevea brasiliensis*) were successfully introduced to south China in the 1950s on a large-scale; however, due to the climate, are prone to cold injury during the winter season. Increased cold tolerance is therefore an important goal, yet the mechanism underlying rubber tree responses to cold stress remains unclear. This study carried out functional characterization of *HbICE1* (Inducer of *CBF* Expression 1) from *H. brasiliensis*. A nucleic protein with typical features of ICEs, HbICE1 was able to bind to MYC recognition sites and had strong transactivation activity. *HbICE1* was constitutively expressed in all tested tissues, with highest levels in the bark, and was up-regulated when subjected to various stresses including cold, dehydration, salinity and wounding. When overexpressed in *Arabidopsis, 35S::HbICE1* plants showed enhanced cold resistance with increased proline content, reduced malondialdehyde (MDA) metabolism and electrolyte leakage, and decreased reactive oxygen species (ROS) accumulation. Expression of the cold responsive genes (*COR15A, COR47, RD29A*, and *KIN1*) was also significantly promoted in *35S::HbICE1* compared to wild-type plants under cold stress. Differentially expressed genes (DEGs) analysis showed that cold treatment changed genes expression profiles involved in many biological processes and phytohormones perception and transduction. Ethylene, JA, ABA, as well as ICE-CBF signaling pathways might work synergistically to cope with cold tolerance in rubber tree. Taken together, these findings suggest that *HbICE1* is a member of the *ICE* gene family and a positive regulator of cold tolerance in *H. brasiliensis*.

## Introduction

Cold stress is one of the most devastating environmental factors adversely affecting plant growth and development, significantly constraining geographic distribution and agricultural productivity. Cold stress interferes with various physiological and biochemical processes via direct inhibition of metabolic reactions and indirect induction of osmotic, oxidative and other stresses. *Hevea brasiliensis*, with its high rate of production and superior rubber quality, is the sole commercial source of natural rubber. A perennial tropical tree species originating from the Amazonian forests of Brazil, rubber trees were traditionally planted within a restricted region between 15° north and 15° south latitudes. Though rubber trees were successfully introduced to south China in the 1950s on a large-scale, they are prone to cold injury during the winter season. Cold stress not only affects rubber production, but also threatens the survival of rubber trees in China. Increased cold tolerance is therefore a major aim of rubber tree breeding programs.

Plants have evolved various physiological, biochemical and molecular strategies aimed at adaptation to adverse situations (Nakashima et al., 2009; Thomashow, 2010; Theocharis et al., 2012). In the past decade, significant progress has been made in deciphering the key components of the cold signaling pathway in the model plants *Arabidopsis* and other species (Wisniewski et al., 2014; Shi Y. et al., 2015). In *Arabidopsis*, three *C-repeat-binding factors* (*CBFs*), *CBF1, CBF2*, and *CBF3*, have been functionally characterized (Jaglo-Ottosen et al., 1998; Medina et al., 1999). Under low temperatures, cold stress rapidly and transiently induces *CBF* expression, stimulating expression of *cold-responsive* (*COR*) genes by binding to C-repeat binding factor (CRT)/dehydration-responsive element (DRE) *cis*-elements in the promoters of *COR* genes, thereby increasing accumulation of proline and total sugar and protecting membranes and proteins from damage (Stockinger et al., 1997; Liu et al., 1998; Thomashow, 1999; Chinnusamy et al., 2007; Maruyama et al., 2012; Shi Y. et al., 2015). ICE1 (inducer of *CBF* expression 1), a constitutively expressed MYC-like bHLH transcriptional activator, also functions during cold acclimation by inducing *CBF* expression via binding of MYC recognition elements in *CBF* promoters (Chinnusamy et al., 2003, 2010; Fursova et al., 2009). The mutant *ice1* was found to exhibit reduced plant tolerance to chilling and freezing stresses due to repression of *AtCBF3* expression and subsequent decreases in expression of various downstream *COR* genes (Chinnusamy et al., 2003; Fursova et al., 2009). In contrast, plants overexpressing either *AtICE1* or *AtICE2* display improved freezing tolerance via enhanced *AtCBF3* and *AtCBF1* expression(Chinnusamy et al., 2003; Fursova et al., 2009). Thus, the ICE-CBF-COR transcriptional regulatory cascade is a well-established plant response to cold stress (Chinnusamy et al., 2007; Shi Y. et al., 2015). Cold acclimation, which is thought to enhance freezing tolerance after exposure to low temperatures (Thomashow, 1999), is one of the major mechanisms of plant adaptation to cold stress. But whether cold acclimation can affect the cold resistance of rubber tree has not been reported so far. Besides transcriptional regulation of ICE gene, several important components have been found to regulate cold acclimation by modulating ICE-CBF pathway at posttranslational levels. The small ubiquitin-related modifier (SUMO) E3 ligase, SIZ1 (SAP and Miz 1) and the RING finger E3 ligase HOS1 (high expression of osmotically responsive genes1) have been shown to modify ICE1 posttranslationally and function in the ICE-CBF/DREB1 signaling pathway (Dong et al., 2006; Miura et al., 2007). Recent reports further suggest that ICE1 is also regulated by various other factors such as jasmonate signaling proteins JAZ 1/4 (JASMONATE ZIM-DOMAIN 1/4), which inhibit ICE1 transcriptional activity (Hu et al., 2013). Moreover, Ding et al. (2015) discovered that the protein kinase OST1 (open stomata 1), a key component in ABA signaling, interacts with and phosphorylates ICE1 protein under cold stress, stabilizing and activating ICE1 and thereby enhancing plant tolerance to freezing temperatures. ICE1 is also degraded by the E3 ligase HOS1 (high expression of osmotically responsive gene 1)-mediated 26S-proteasome pathway (Dong et al., 2006). More recently, Huang X. S. et al. (2015) reported that ICE1 from *Poncirus trifoliate* functions in cold tolerance by altering polyamine accumulation via interaction with arginine decarboxylase. ICE1 is therefore not only a central component in cold signaling, but also serves as a convergence point, integrating signals to regulate cold tolerance in plants.

Studies on cold stress in rubber trees were mainly focused on changes in physiological parameters and alleviating injury after cold stress, the major components of cold signaling of rubber tree were nearly unidentified except *HbCBF1*and the molecular mechanisms underlying how rubber tree responses to cold stress were still very poorly understood (Cheng et al., 2015). In this study, the *H. brasiliensis ICE1* (*HbICE1*) gene was cloned and functionally characterized for its role in cold tolerance, revealing its location in the nucleus and ability to bind to MYC recognition sites. Overexpression of *35S::HbICE1* in *Arabidopsis* enhanced cold resistance probably due to increased proline level, reduced MDA content and electrolyte leakage, and decreased reactive oxygen species (ROS) accumulation under cold stress. Furthermore, the cold responsive genes (*COR15A, COR47, RD29A*, and *KIN1*) were significantly activated in overexpressing plants compared to the wild-type (WT) under cold conditions. Taken together, these data suggest that *HbICE1* is a functional member of the *ICE* gene family, playing a positive role in cold resistance in *H. brasiliensis*.

## Materials and methods

### Plant material and treatments

Reyan 7-33-97 rubber trees (*H. brasiliensis*) cultivated at the experimental plantation of Hainan University, Hainan Province, China, were used in this study. The plants were pruned annually. To examine tissue-specific expression of *HbICE1*, samples from six tissues (the latex, leaves, stem, bark, stamen and pistil) were collected for RNA extraction from 17-year-old mature trees tapped for the previous two years. To determine expression of *HbICE1* in response to NaCl and dehydration treatment, four batches of 12 seedlings per treatment were selected. Three batches were treated with 200 mM NaCl or 10% polyethylene glycol (PEG), and one with ddH_2_O then the leaves collected for RNA isolation. For cold treatment, seedlings were transferred to a culture room at 4°C under a 12 h light/12 h dark cycle with 80% humidity for 1, 3, 6, 12, or 24 h then the leaves collected for RNA isolation. For wounding treatment, leaves of 5-year-old mature virgin (untapped) trees were wounded with a hemostat then the leaves collected for RNA extraction.

To generate *35S::GFP-HbICE1* transgenic *Arabidopsis* plants, the coding region of *HbICE1* was amplified using primers 5′-gaattcATGCTTGATACCGACTGGTATGATA-3′and 5′-gaattcTCACATCATTCCATGAAAGCCAGCT-3′. The coding sequence fragment was then subcloned into the *ECOR1* site of the pEGAD vector and the recombinant plasmid introduced into *Arabidopsis* ecotype *Col-0* via *Agrobacterium tumefaciens* (GV3101)-mediated transformation using the floral dip method (Clough and Bent, 1998). *Arabidopsis* plants were sown in vermiculite in pots at 23°C under 16 h light/8 h dark conditions with 75% humidity and a light intensity of 150 mmol m^−2^ s^−1^.

To determine cold tolerance of the transgenic plants, a cold treatment assay was performed as described previously with slight modifications (Ding et al., 2015). Fourteen-day-old seedlings cultivated in 1/2 MS medium at 23°C were transferred to 4°C for 2 days then exposed to −8°C for 4 h and subsequently returned to normal conditions. Survival rates were measured after 7 days. To determine cold tolerance of the rubber tree seedlings, seedlings with or without cold acclimation (1 day at 4°C) were exposed to −16°C for 0.5 and 1 h and subsequently returned to normal conditions. The freezing treatment experiment was performed in triplicate. For *Arabidopsis*, T3 or T4 homozygous transgenic plants were used in this study.

### Isolation and bioinformatics analysis of *HbICE1*

The CDS of HbICE1 was predicted using Bioedit software (http://www.mbio.ncsu.edu/BioEdit/bioedit.html) and confirmed using BLASTP on the NCBI BLAST server (http://blast.ncbi.nlm.nih.gov/Blast.cgi). The detailed *Insilico* cloning procedure was carried out as previously described (Hong et al., 2015). Molecular weights (MW) and isoelectric points (pI) were predicted using ExPASy (http://www.expasy.org/tools), nuclear localization signals were predicted using the online server (http://www.predictprotein.org/), and protein secondary domains were predicted with a Motif scan (http://myhits.isb-sib.ch/cgi-bin/motif_scan). Sequence alignment was performed using DNAMAN software and a phylogenetic tree constructed using MEGA 5.1 software.

### Subcellular localization of HbICE1

The full-length CDS of HbICE1 was fused to the C terminus of the green fluorescent protein (GFP) of the pEGAD vector, driven by the 35S promoter. GFP in the roots of homozygote transgenic *Arabidopsis 35S::GFP-HbICE1* plants was examined using confocal microscopy (excitation and emission: 488 and 515 nm, respectively; 22°C).

### Transcriptional activation assay and MYCR-binding assay of HbICE1

For the transcriptional activation assay, the open reading frame (ORF) of HbICE1 was amplified by PCR using primers P1 (cgGAATCCatgcttgataccgactggta) and P2 (aaCTGCAGcatcattccatgaaagccag). The coding sequence fragment was then subcloned into the *EcoR*I and *Pst*I restriction sites of the pGBKT7 vector, giving pGBKT7-HbICE1. The recombinant vector pGBKT7-HbICE1, as well as pGBKT7-53+pGADT7-T (positive control) and pGBKT7 (negative control), were transformed into yeast strain AH109 according to the manufacturer's instructions (Clontech, PT4084-1). The transformed yeast was placed on plates containing SD/-Trp-Leu/X-α-Gal/Aureobasidin A, SD/-Trp-Leu, SD/-Trp, SD/-Trp-His or SD/-Trp-His-Ade medium then incubated at 28°C for 3–4 days for analysis of transformant growth.

To investigate whether HbICE1 was able to bind to the MYC recognition sequence, a yeast one–hybrid experiment (Y1H) was performed according to the manufacturer's instructions (Clontech). The ORF of HbICE1 was fused to the pGADT7 vector digested with *EcoR*I to create pGADT7-HbICE1. A 66-bp DNA fragment containing triple tandem repeats of the sequence containing the MYC recognition sequence (CACATG) was inserted into the pHIS2 vector, generating the recombinant vector pHIS2-MYCR. pHIS2-MYCR was then transformed into yeast strain Y187 on plates containing SD/-Trp, SD/-Trp/-His or SD/-Trp/-His/10mM 3-AT medium to verify autoactivation of pHIS2-MYCR. Both pGADT7-HbICE1 and pHIS2-MYCR were subsequently co-transformed into Y187 to verify the DNA sequence MYCR and HbICE1 protein interactions. The transformed yeast strains were placed on plates containing SD/-Trp/-Leu, SD/-Trp/-Leu/-His, or SD/-Trp/-Leu/-His/10mM 3-AT medium then incubated at 28°C for 3–4 days for analysis of transformant growth.

### RNA extraction and quantitative real-time PCR

*Hevea* RNA was extracted from leaves of *H. brasiliensis* as described previously (Xia et al., 2011). *Arabidopsis* RNA was extracted using Trizol reagent (Invitrogen, Carlsbad, CA, USA) according to the instruction manual. All RNA samples were treated with RNase-free DNase I (Promega, Madison, WI, USA) to digest genomic DNA. The concentration and quality of DNaseI-treated total RNA was determined by spectrophotometry and agarose gel electrophoresis. As a template for first-strand cDNA synthesis, 2 μg DNase I-treated RNA was used according to the manufacturer's instructions (RevertAid First Stand cDNA Synthesis kit; Fermentas, Vilnius, Lithuania). Quantitative real-time PCR (qRT-PCR) was carried out using ABI-7500 Real-Time PCR apparatus with SYBR Green I dye (Takara, Tokyo, Japan) as follows: 95°C for 3 min followed by 40 cycles of 95°C for 20 s, 58°C for 15 s and 72°C for 20 s. The efficiency of each primer pair was evaluated prior to PCR using the primers listed in Table S1. Relative levels were calculated as 2^−ΔΔCT^. Each biological sample was performed with three technical repetitions, and data analysis carried out using three independent biological replicates. The whole process from plant material treatment, RNA extraction, cDNA synthesis to qRT-PCR was repeated three times per treatment as three independent biological replicates. Values were statistically analyzed by ANOVA or the Student's *t*-test.

### Analysis of malondialdehyde (MDA) and proline contents, and electrolyte leakage

Three-week-old seedlings of transgenic *35S::HbICE1* and WT *Arabidopsis* were subjected to cold stress treatment then leaves harvested to determine MDA and proline contents, and the degree of electrolyte leakage. Proline accumulation was determined as described previously (Shi H. T. et al., 2012). Briefly, 0.25 g leaf samples were harvested and extracted in 3% sulfosalicylic acid then centrifuged at 12,000 × g for 10 min. The supernatant (2 mL) was incubated with 2 mL ninhydrin reagent [2.5% (w/v) ninhydrin, 60% (v/v) glacial acetic acid, and 40% 6M phosphoric acid] and 2 mL glacial acetic acid at 100°C for 40 min, and the reaction terminated in an ice bath. MDA levels were measured using the thio-barbituric acid (TBA) method as described previously (Cai et al., 2015). Electrolyte leakage was measured as described previously (Lin et al., 2012). Eight leaves from transgenic and WT plants treated with and without cold treatment were placed in a bottle with 40 mL ddH_2_O_2_, shaken at 120 rpm for 3 h then conductivity (C1) measured using an ion leakage meter. Conductivity (C2) was measured after boiling the leaves for 30 min and shaking for 1 h. Electrolyte leakage was calculated as (C1/C2) × 100%.

### Screening of differentially expressed genes (degs) based on RNA-seq

Total RNA was isolated from the rubber tree seedlings treated at 4°C for 3 and12 h, including three biological repeats for each condition. RNA-Seq was performed by the Beijing Genomics Institute (Shenzhen, China). Oligo (dT) magnetic beads were used to select mRNA with polyA tail, followed by DNase I reaction to remove DNA probe. The purified mRNA was used reverse transcription to double-strand cDNA (dscDNA) by N6 random primer. End of dscDNA was repaired with phosphate at 5′ end and stickiness “A” at 3′ end, then ligated and with adaptor. Two specific primers of adaptor were used to amplify the ligation product. The PCR product was denatured by heat and the single strand DNA was cyclized by splint oligo and DNA ligase. The prepared library was sequenced by (Illumina HiSeqTM 2000).

Clean reads were mapped to the *hevea* contigs assembly using SOAPaligner/soap2 mismatches; no more than 2 bases were allowed in the alignment. The number of clean reads for each gene was calculated and then normalized to Reads Per Kb per Million reads (RPKM), which associates the read number with the gene expression levels. Further, deep analysis were performed based on DEGs, including Gene Ontology (GO) enrichment analysis, KEGG pathway enrichment analysis, cluster analysis, protein-protein interaction network analysis and finding transcription factor.

## Results

### Cloning and bioinformatics analysis of *HbICE1*

**Figure 1:**
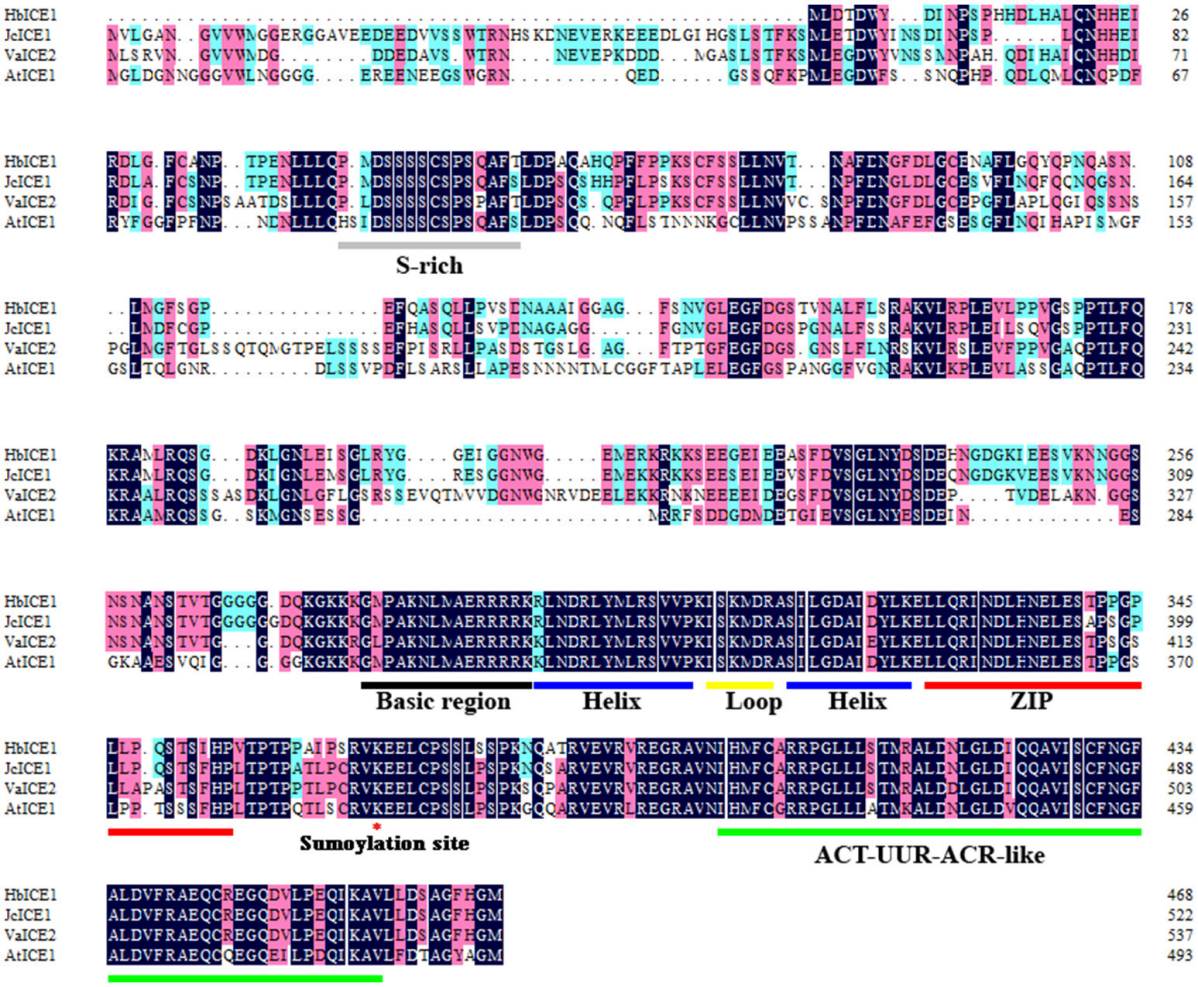
Amino acid sequence alignment of HbICE1 and ICEs from other plant species. Sequences and accession numbers are as follows: *Jatrophacurcas* (NP_001306848.1), *Vitisamurensis* (AGP04218.1) and *Arabidopsis thaliana* (NP_189309.2). Blue and pink backgrounds indicate identical and similar residues, respectively. The S-rich motif, basic-helix-loop-helix-leucine zipper (bHLH-ZIP) region, ACT-UUR-ACR-like domain, and sumoylation site are labeled.

**Figure 2:**
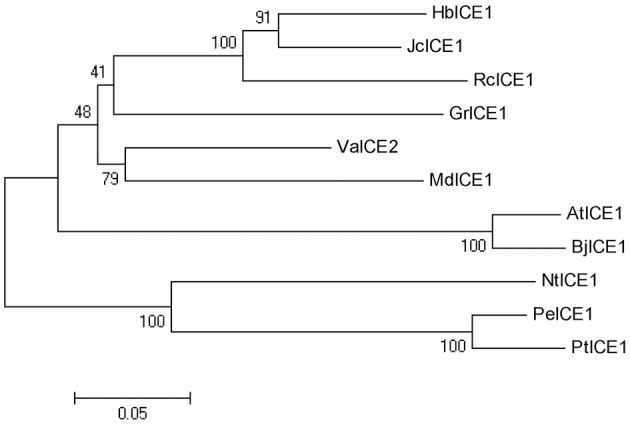
Phylogenetic analysis of HbICE1 and ICEs from other plant species. The neighbor-joining method was used to construct the tree. GenBank accessions of the predicted ICE protein sequences used are as follows: JcICE1 (NP_001306848.1), VaICE1 (AGP04218.1), AtICE1 (NP_189309.2), RcICE1 (XP_015570780.1), GrICE1 (XP_012489464.1), MdICE1 (XP_008379053.1), PeICE1 (XP_011040262.1), PtICE1 (XP_002318166.1), NtICE1 (XP_009625133.1) and BjICE1 (AEB97375.2).

Plant *ICE1*-like genes play a critical role in cold tolerance in a number of different plants; however, the *ICE1* gene has yet to be identified in *H. brasiliensis*. To clone the *HbICE1* gene from *H. brasiliensis*, the *A. thaliana* ICE1 protein sequence was used as a query sequence in a blast search against the *Hevea* EST and nucleotide database. The matching sequence with the lowest *E*-value was subsequently selected as a backbone for further *insilico* assembly of full-length cDNA. The predicted full-length HbICE1 cDNA contained an ORF of 1410 bps, as validated by PCR amplification and sequencing. The predicted ORF encoded a protein of 469 amino acid residues with an estimated MW of 51 kDa and a pI of 5.30. Multiple alignments of HbICE1 and ICE1 proteins from other plants indicated that the C terminus of all ICE1 proteins tested was highly conserved, whereas the N terminus varied (Figure 1). Furthermore, HbICE1 contained a MYC-like basic helix-loop-helix (bHLH) domain, a serine-rich region (S-rich), a zipper region (ZIP) and an ACT-UUT-ACR like domain, all of which are typical features of ICE proteins (Figure 1). A phylogenetic tree was reconstructed using the deduced amino acid sequence of HbICE1 and other plant ICEs, revealing that HbICE1 is most closely related to JcICE1 of *Jatrophacurcas* (Figure 2).

### HbICE1 is nuclear-localized and has transactivation activity in yeast

**Figure 3:**
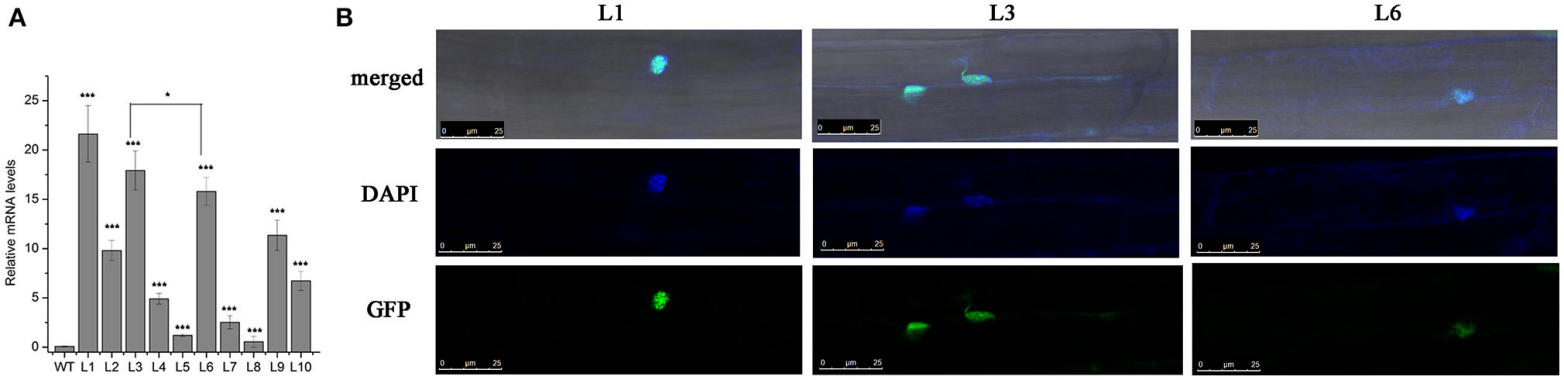
Subcellular localization of the HbICE1 protein. **(A)** Confirmation of *HbICE1* expression in selected transgenic lines. mRNA levels of *35S::GFP-HbICE1* lines and the wild–type (WT) were examined by qRT–PCR analysis, using *AtEIF4* as the internal standard. **(B)** Representative images show root cells of 5-day-old *35S::GFP-HbICE1* transgenic *Arabidopsis* seedlings. DAPI was used to stain nuclei (pseudo-color, blue).

As a possible transcriptional factor, HbICE1 should be located in the nucleus for transcription regulation. To verify this, we created a construct expressing the GFP-HbICE1 fusion protein by fusing GFP in-frame to the 5′ end of the HbICE1 ORF under control of the cauliflower mosaic virus 35S promoter then transforming the construct into WT *Arabidopsis* using the floral dip method. Following selection by Basta resistance, independent transgenic lines were selected for verification by genomic PCR and qRT-PCR analysis. QRT-PCR data revealed high expression of *HbICE1* in six lines (L1, L2, L3, L6, L9, and L10), three of which (L1, L3, and L6) showing highest transcript levels were selected for further analysis (Figure 3A). Roots of homozygote *35S::GFP-HbICE1* plants were examined using confocal microscopy revealing exclusive expression of the GFP-HbICE1 protein in the nucleus (Figure 3B). This finding confirmed that HbICE1 is a nuclear protein, consistent with a previous report suggesting that *Arabidopsis* ICE1 is a transcription factor (Chinnusamy et al., 2003).

**Figure 4:**
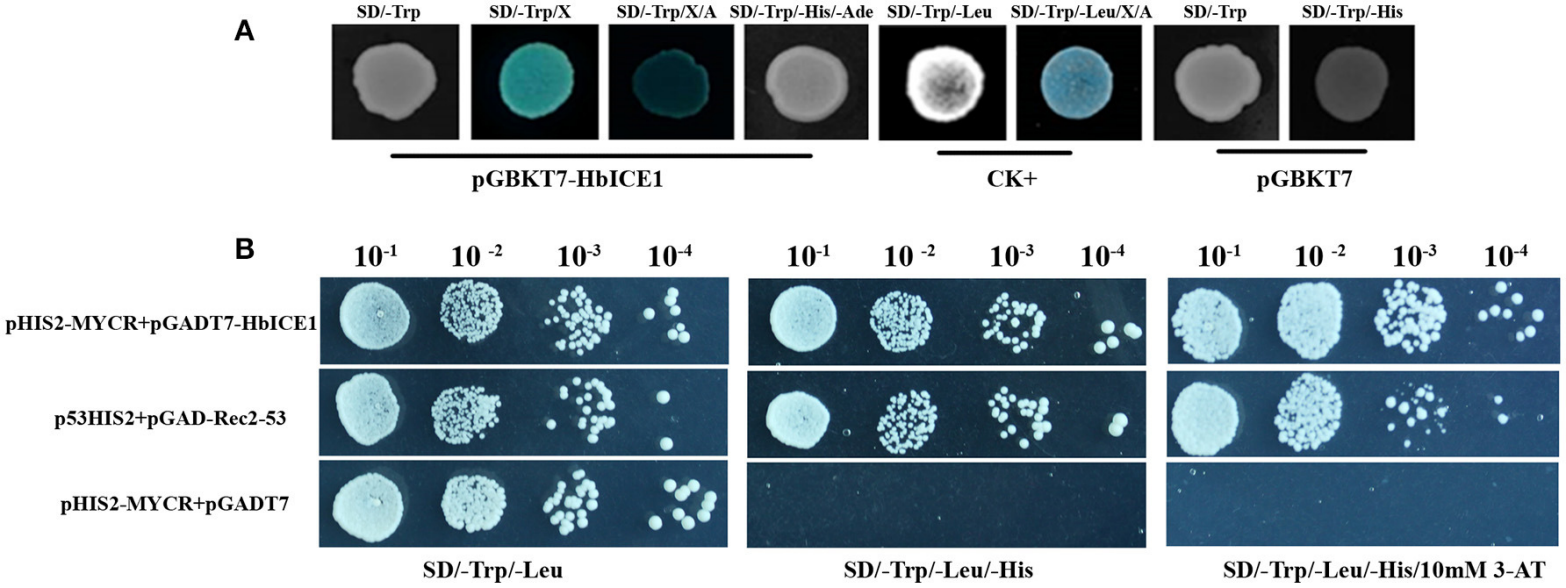
Transcriptional activation assay of HbICE1. **(A)** Growth of AH109 yeast cells transformed with vectors of the positive control (pGBKT7-53 and pGADT7-T, CK+), negative control (pGBKT7), and pGBKT7-*HbICE1* on various selection media. SD/-Trp-Leu/X-α-Gal/Aureobasidin A medium is indicated by SD/-Trp-Leu/X/A. **(B)** Growth of Y187 yeast cells co-transformed with vectors of the positive control (p53HIS2+pGAD-Rec2-53), negative control (pHIS2-MYCR+pGADT7), and pGADT7-HbICE1 with pHIS2-MYCR on SD/-Leu-Trp and SD/-Leu-Trp-His selection medium with and without 10 mM 3-AT.

Next, we assayed transactivation activity of HbICE1, another important feature of transcription factors. As shown in Figure 4A, yeast transformed with both pGBKT7-53 and pGADT7-T (CK+) grew well on SD/-Trp-Leu and SD/-Trp-Leu/X-α-Gal/Aureobasidin A medium. Moreover, while yeast cells transformed with pGBKT7-*HbICE1* grew well on SD/-Trp medium, they grew normally on SD/-Trp-His-Ade medium, exhibiting fairly strong β-galactosidase activity. In contrast, yeast cells transformed with the negative control (pGBKT7) did not grow on SD/-Trp-His medium (Figure 4A). These results further confirm that HbICE1 has transcriptional activation activity.

Since *Arabidopsis* ICE1 can bind to the MYC-recognition element (Chinnusamy et al., 2003), we also explored whether HbICE1 could bind to a sequence containing the MYC recognition element using a yeast one hybrid (Y1H) assay. As shown in Figure 4B, yeast cells co-transformed with pHIS2-MYCR and pGADT7-HbICE1 grew as well as those containing the positive control (p53HIS2+pGAD-Rec2-53) on SD/-Trp-Leu-His medium with and without 10 mM 3-AT. In contrast, cells co-transformed with the negative control (pHIS2MYCR+pGADT7) did not grow under this condition, further suggesting that HbICE1 binds to MYC recognition sites, activating the transcription of report genes in yeast.

### Analysis of *HbICE1* expression patterns

**Figure 5:**
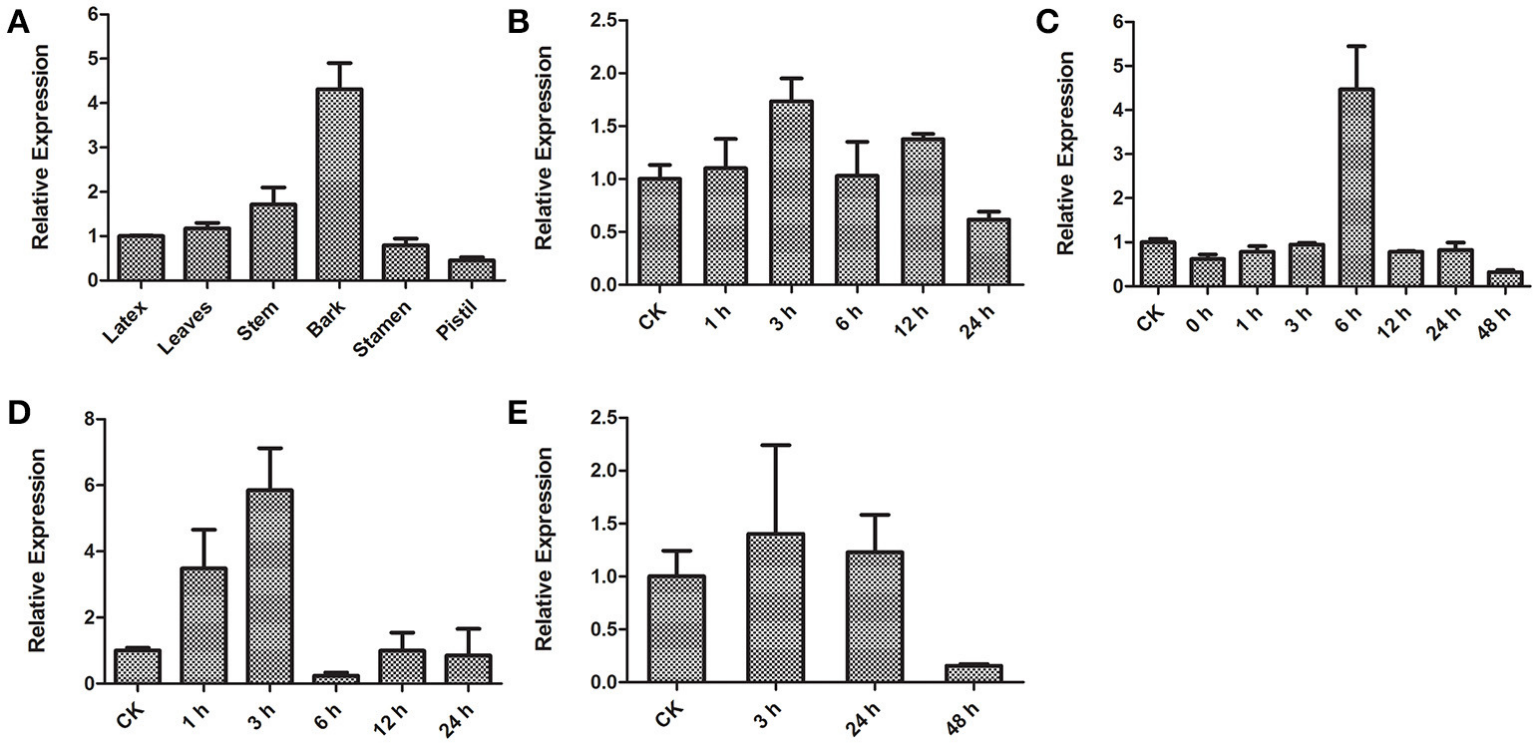
Transcription patterns of *HbICE1* determined by qRT-PCR. **(A)** Differential expression of *HbICE1* in various tissues (the latex, leaves, stem, bark, stamen and pistil). Time-course expression patterns of *HbICE1* in response to different biotic and abiotic stresses: cold **(B)**, dehydration **(C)**, wounding **(D)**, and salt stress **(E)**.

To investigate tissue-specific expression of *HbICE1, HbICE1* expression in the latex, leaves, stem, bark, stamen and pistil was examined by qRT-PCR. *HbICE1* was universally expressed in all tissues tested with the highest expression in bark (Figure 5A). We also examined expression profiles of *HbICE1* under various abiotic stresses including cold, dehydration, wounding, and salinity. *HbICE1* was significantly induced at 3 h after cold treatment, but expression decreased to a low level at 24 h (Figure 5B). Under dehydration, *HbICE1* mRNA was markedly induced by nearly 4.5-fold at 6 h then quickly returned to the normal level at 12 h post treatment (Figure 5C). After wounding treatment, the *HbICE1* transcripts showed strong induction at 1 and 3 h (Figure 5D), while under salt stress, expression was induced 3 h after treatment (Figure 5E). These results indicate that *HbICE1* expression is regulated during plant responses to multiple abiotic stresses.

### Over-expression of *HbICE1* in *Arabidopsis* increases cold tolerance

**Figure 6:**
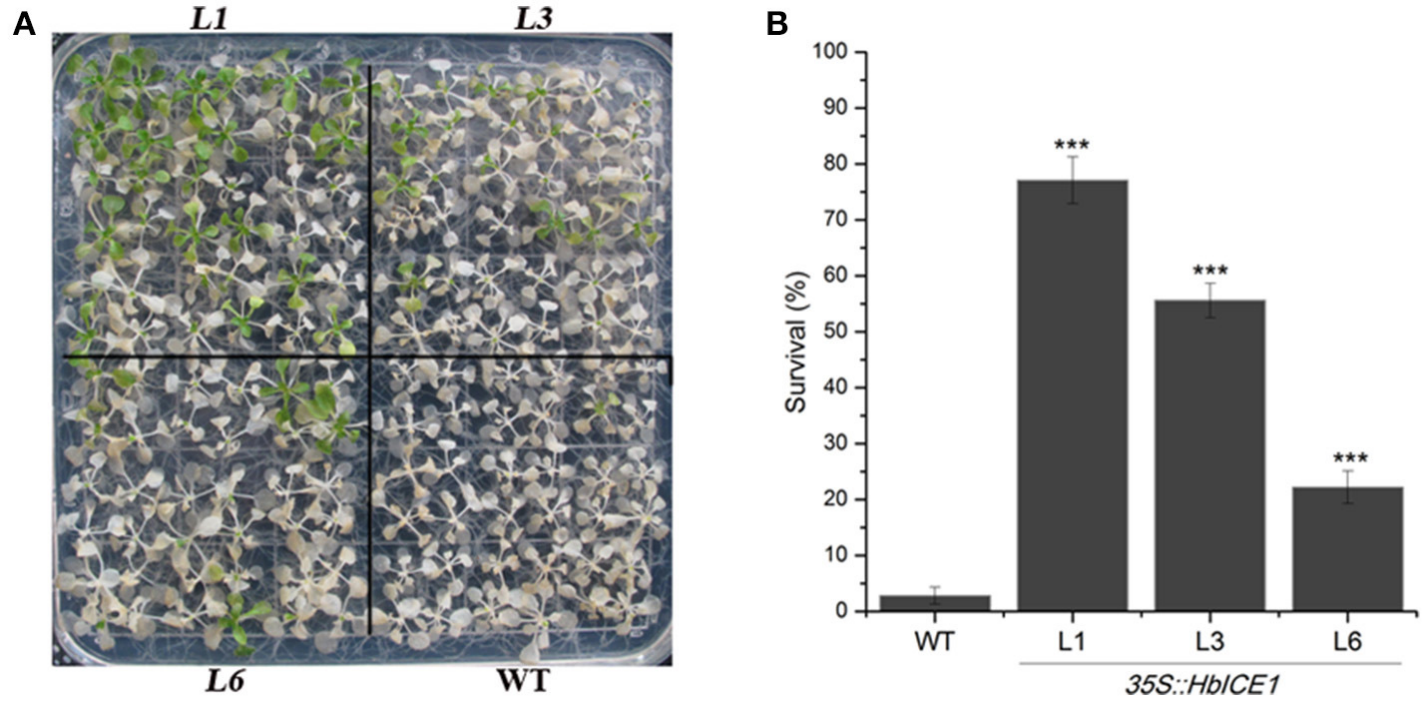
Overexpression of *HbICE1* confers enhanced cold tolerance in *Arabidopsis*. Freezing phenotypes **(A)** and survival rates **(B)** of transgenic lines L1, L3, and L6 and the WT. Two-week-old *Arabidopsis* plants were transferred to 4°C for 2 days, exposed to −8°C for 4 h then returned to normal conditions. Photographs were taken before and after 7-d recovery. In **(B)**, data represent the means of three replicates ± SD. Asterisks indicate significant differences compared with the WT under the same treatment condition (^***^P < 0.005, student's *t*-test).

To further determine the role of *HbICE1* in cold tolerance, *HbICE1-*overexpressing lines and WT plants were subjected to freezing treatment. As shown in Figure 6A, *HbICE1-*overexpressing lines displayed less freezing damage and increased survival ratecompared with the WT after 1-week recovery. The survival rate of WT plants was only 2.8%, significantly lower than that of the transgenic lines (77.1% for L1, 55.6% for L3, and 22.2% for L6; Figure 6B), suggesting that overexpression of *HbICE1* conferred cold tolerance in transgenic *Arabidopsis*.

### Over-expression of *HbICE1* affects proline content, electrolyte leakage, MDA metabolism and H_2_O_2_ accumulation under cold stress

**Figure 7:**
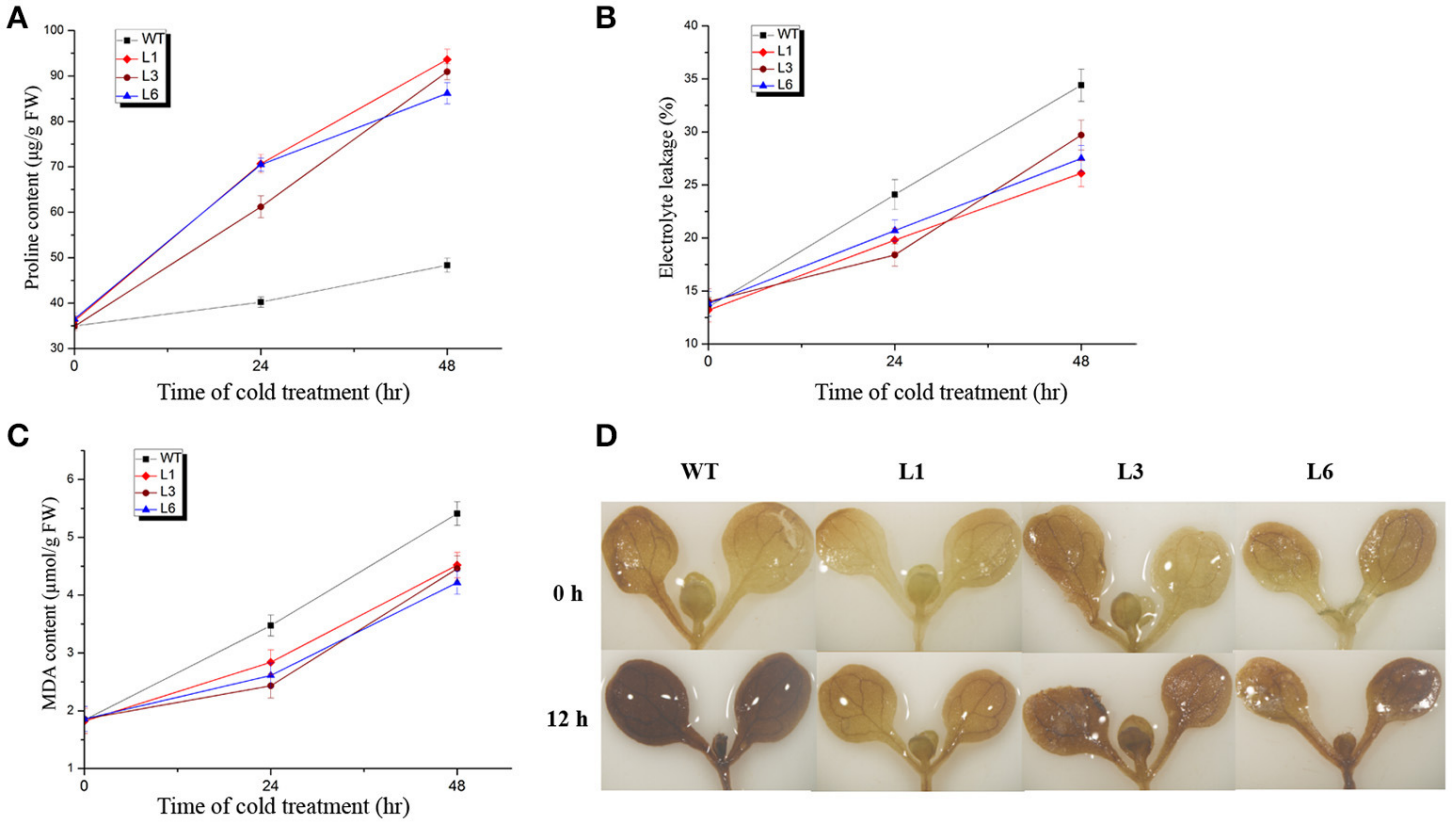
Changes in physiological parameters of *35S::GFP-HbICE1* lines and wild-type (WT) plants under cold stress. Three-week-old *Arabidopsis* plants were grown at 0°C for the time indicated then leaves collected to determine the free proline content **(A)**, electrolyte leakage **(B)** and MDA content **(C)**. In **(A–C)**, data represent the mean ± SD (*n* = 3). **(D)** DAB staining of 6-day-old *Arabidopsis* plants with and without cold treatment.

Many physiological parameters such as proline content, electrolyte leakage, MDA metabolism and ROS accumulation are known indicators of tolerance to a wide variety of abiotic stresses (Feng et al., 2012; Xu et al., 2014; Cai et al., 2015; Huang X. S. et al., 2015). Thus, these physiological parameters were subsequently examined in *HbICE1*-overexpressing lines under cold stress. Proline accumulation is considered an adaptive response to various kinds of environmental stress, conferring stress tolerance by promoting osmotic adjustment, protecting membranes and proteins, and inhibiting ROS production. Under normal growth conditions, the proline content of the *HbICE1*-overexpressing lines was similar to that of the WT. However, after 24 and 48 h at 0°C, levels were significantly higher in the transgenic lines compared to the WT. At 48 h after treatment, proline levels were 2-fold higher in the transgenic compared to the WT plants (Figure 7A).

Electrolyte leakage is another reliable indicator of cell membrane damage during the plant stress response (Feng et al., 2012; Huang X. et al., 2015; Cheng et al., 2016). Under normal growth conditions, the transgenic lines and WT plants showed similar levels of electrolyte leakage ranging from 13 to 15%. However, during cold treatment, electrolyte leakage was considerably less in the transgenic lines compared to the WT, suggesting a reduction in cell membrane damage in the *HbICE1*-overexpressing lines (Figure 7B), and therefore, improved cold tolerance. MDA is also an important index of plant oxidative stress and cell injury in response to stress conditions. While no significant differences in MDA content were observed between transgenic lines and WT plants under normal conditions, lower values were observed in the transgenic compared to WT plants after cold treatment (Figure 7C).

ROS-induced oxidative damage is associated with much of the stress-induced damage that occurs at the cellular level (Chinnusamy et al., 2007). Stress-induced ROS production results in degradation of polyunsaturated lipids, resulting in formation of MDA. In other words, the reduction in MDA content in the transgenic plants might reflect a decrease in ROS accumulation under cold stress. We therefore assayed ROS accumulation in the *35S::HbICE1* plants using DAB staining. As expected, stronger staining was observed in the WT plants compared with the transgenic plants under cold conditions (Figure 7D), suggesting higher levels of damage in the WT. Taken together, these findings suggest that the increased cold tolerance of *35S::HbICE1* plants is the result of increased proline accumulation, reduced electrolyte leakage and MDA metabolism, and a decrease in H_2_O_2_ accumulation under cold conditions.

### *HbICE1* positively regulates cold-responsive gene expression under cold conditions

**Figure 8:**
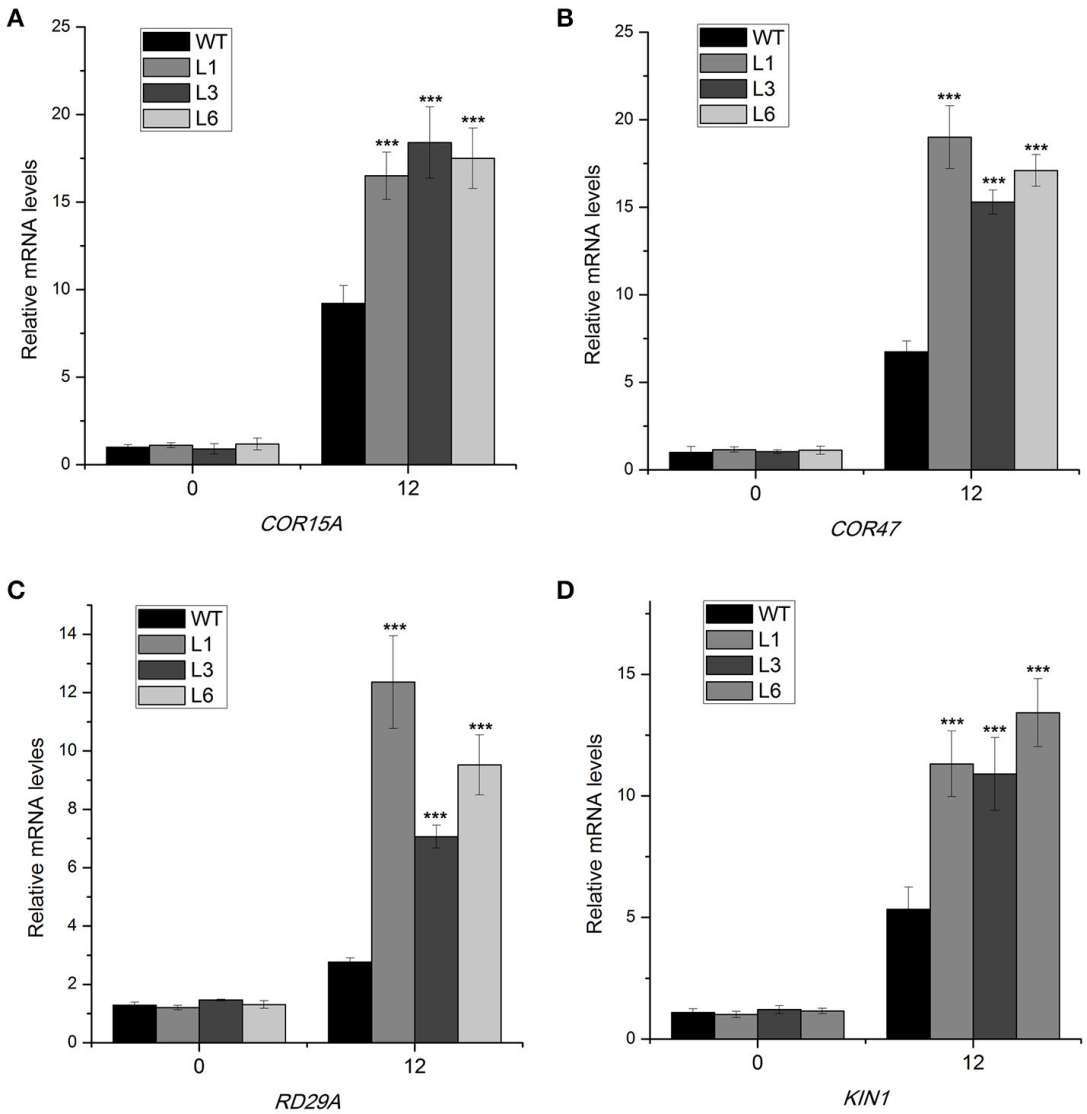
Expression of cold-responsive genes in *HbICE1*-overexpressing lines subjected to cold stress. Expression of *COR15A* **(A)**, *COR47* **(B)**, *RD29A* **(C)**, and *KIN1***(D)** in wild-type (WT) and *HbICE1*-overexpressing lines under normal and cold conditions assayed by quantitative real-time PCR, using *AtEIF4* as the internal standard. Data represent means of three replicates ± SE, and asterisks indicate significant differences at ^***^P < 0.005 (Student's *t*-test).

To further elucidate the molecular mechanism of the *35S::HbICE1* plant response to cold stress, we examined transcript levels of cold-responsive genes using qRT-PCR. These cold response genes (*COR15A, COR47, KIN1*, and *RD29A*), which contain DRE or related motifs, are downstream target genes of CBF (Stockinger et al., 1997). Under normal conditions, expression levels of *COR15A, COR47, KIN1*, and *RD29A* were relatively low in both the transgenic and WT plants. In contrast, after cold exposure for 12 h, expression levels of all tested genes were significantly up-regulated in all three *35S::HbICE1* lines compared with the WT (Figures 8A–D), suggesting that overexpression of HbICE1 positively regulates cold-responsive gene expression, thereby contributing to improved cold tolerance.

### Analysis of differentially expressed genes (DEGs) based on RNA-seq of rubber tree in response to cold stress

**Figure 9:**
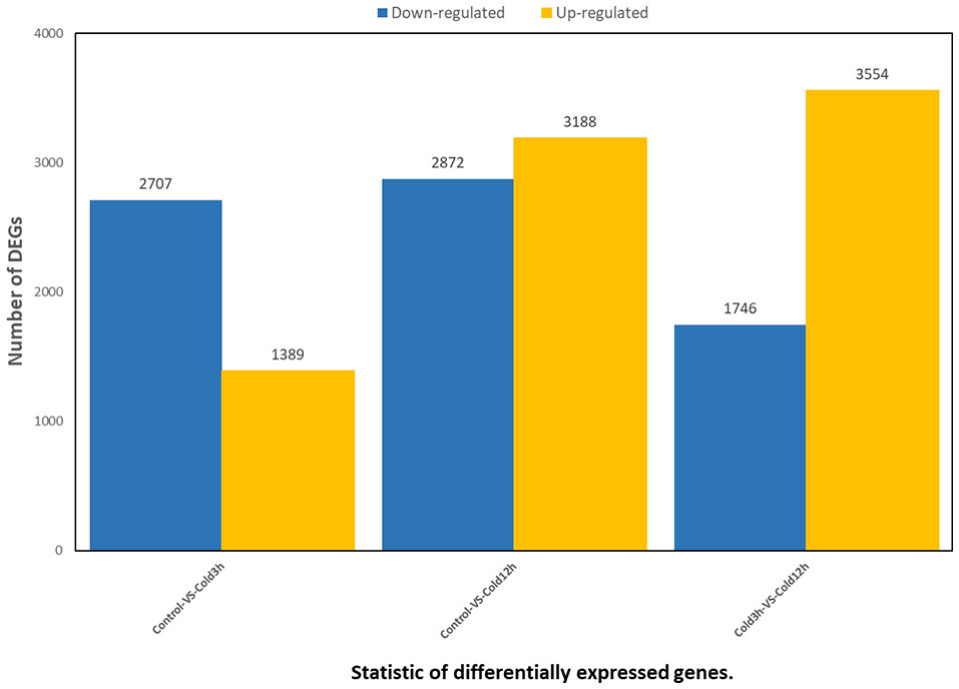
Statistic of differentially expressed genes. X axis represents pairwise and Y axis means number of screened DEGs. Blue bar denotes down-regulated genes and orange bar for the up-regulated.

In order to gain a global view of transcript expression in rubber tree in response to cold stress, RNA-Seq was used to analyze the differentially expressed genes, generating 24,108,424 raw sequencing reads and 24,050,817 clean reads after filtering low quality (Table S2). 96.50% reads of the control samples matched to a unique or multiple genomic locus, whereas 96.60% of the 3 h cold treatment sample and 96.16% of 12 h cold treatment matched, respectively (Table S3). A total of 8077genes showed differential expression (low false discovery rate [FDR] < 0.001 and *P*-value < 0.05), including 4096 genes at 3 h (1389 up-regulated, 2707 down-regulated) and 6060 genes at 12 h (3188 up-regulated, 2872 down-regulated) after cold treatment (Figure 9, Tables S4, S5). More DEGs appeared at 12 h than at 3 h after treatment. All the DEGs could be categorized into three main clusters, e.g., biological process, cellular component and molecular function according to GO classification. In the cluster of “biological process,” 17 GO terms of were significantly enriched, of which the GO terms “metabolic process,” “single-organism process,” “cellular process,” “location,” “response to stimulus” were most evidently enriched (Figure S1), suggesting that the these biological processes were responsive to cold stress in rubber tree.

### Pathways enrichment analysis of DEGs

**Figure 10:**
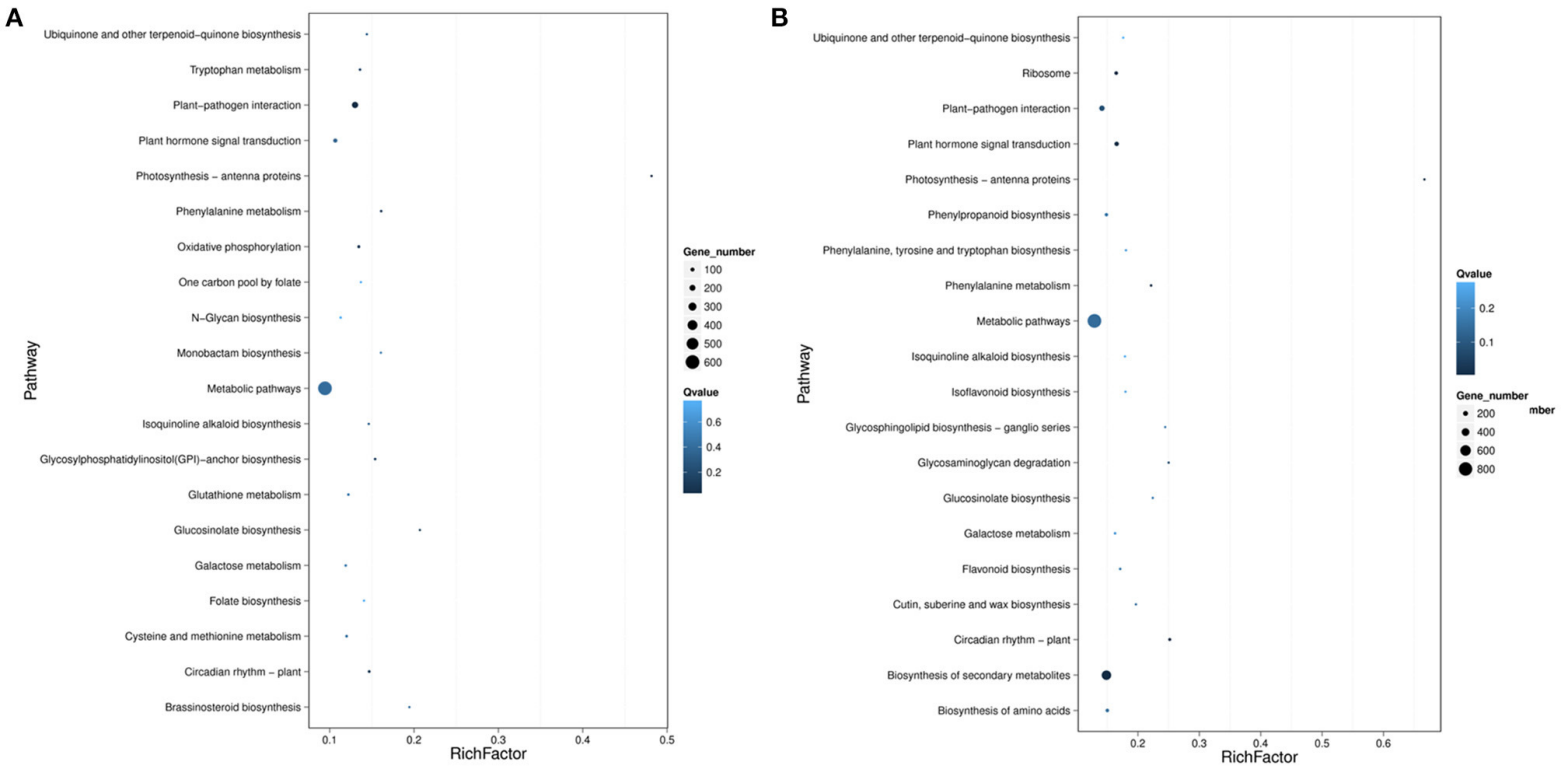
Statistics of pathway enrichment of DEGs in pairwise of Control-VS-Cold3 h **(A)** and Control-VS-Cold12 h **(B)**. Rich Factor is the ratio of differentially expressed gene numbers annoted in this pathway term to all gene numbers annoted in this pathway term. Greater rich Fator means greater intensiveness. *Q*-value is corrected *p*-value ranging from 0 to 1, and less *Q*-value means greater intensiveness. We just display the top 20 of enriched pathway terms.

To further reveal the biological functions of cold responsive genes in rubber tree, pathway enrichment analysis of DEGs based on KEGG database were performed, generating a scatter plot of the top 20 of KEGG enrichment (Figure 10). Most DGEs were enriched in “metabolic pathway” and “biosynthesis of secondary metabolites.” Interestingly, “plant-pathogen interaction,” and “plant hormone signal transduction” pathways were also significantly enriched. Noteworthy, the DGEs enriched in “plant hormone signal transduction” pathway showed most differentially expression in both samples, suggesting that phytohormones involved in the perception and transduction of cold induced signaling in rubber tree. In total, 50 DEGs associated with biosynthesis and/or signal transduction of the phytohormones, including 13 genes related with auxin, 13 genes related with ethylene, 9 genes related with gibberellin (GA), 9 genes related with jasmonic acid (JA), 4 genes related with abscisic acid (ABA) and 2 genes related with cytokinin (CK) (Table 1). Interestingly, ABA 8′-hydroxylase 2 (scaffold0153_318575), ABSCISIC ACID-INSENSITIVE 5-like protein 2 isoform X1 (scaffold0430_516715), and two ABA receptors (scaffold2344_2623, scaffold0748_467762), were down-regulated by cold treatment at both 3 h and 12 h. Furthermore, 12 ethylene-responsive transcription factors (ERFs) showed differential expression, of which nine *ERFs* (scaffold0359_512732, scaffold0636_609282, scaffold0668_410096, scaffold0668_421116, scaffold0770_505198, scaffold0770_519202, scaffold0782_27868, scaffold1267_104008, scaffold2594_1826) were up-regulated and three ERFs (scaffold0024_3294824, scaffold0447_369296, scaffold1195_120325) were down-regulated at 12 h after cold treatment. Additionally, four genes related with JA biosynthesis, two encoding allene oxide cyclase (AOC, scaffold1038_209563, scaffold1142_120639), one encoding 12-oxophytodienoate reductase (OPR, scaffold0150_641) and one encoding latex allene oxide synthase (AOS, scaffold1632_19091), were up regulated at 12 h after cold treatment. Furthermore, three *JAZ* genes and one *MYC2* were also significantly up-regulated, strongly suggesting that JA signaling pathway involved in cold response of rubber tree.

**Table 1:** Hormone-related genes that were differentially expressed during the cold treatment.

	**Expression levels (log2ratio[cold/control])**		**Annotation**
	**Control-vs.-Cold 3 h**	**Control-vs.-Cold 12 h**	
**AUXIN**			
scaffold0198_1391731	−1.7	1.4	Auxin-responsive protein IAA27-like [Jatropha curcas]
scaffold0369_43127	−1.0	−1.8	Auxin-induced protein X10A like [Vitis vinifera]
scaffold0425_327131	0.7	2.5	Auxin-binding protein ABP19a like [Ricinus communis]
scaffold0475_945027	2.6	2.2	Auxin-induced protein 6B like [Vitis vinifera]
scaffold0548_182005	−2.5	1.4	Auxin-responsive protein IAA14 like [Jatropha curcas]
scaffold0933_193093	2.0	2.2	Auxin-binding protein ABP20 like [Populus trichocarpa]
scaffold4789_3384	1.8	1.5	Auxin-responsive protein IAA29 [Jatropha curcas]
scaffold0064_497215	−0.1	1.2	Auxin-responsive protein IAA1 [Jatropha curcas]
scaffold0239_815404	0.3	1.5	Auxin-responsive protein IAA16-like [Populus euphratica]
scaffold0319_1118315	0.4	1.5	Auxin-induced protein AUX28 [Ricinus communis]
scaffold0375_663805	−0.6	−2.0	Auxin response factor 1-like [Populus euphratica]
scaffold1315_157576	0.1	1.3	Auxin-responsive protein IAA9 [Jatropha curcas]
scaffold1418_119368	−0.4	−1.2	Auxin signaling F-BOX 2-like [Jatropha curcas]
**ETHYLENE**			
scaffold0024_3294824	1.5	−1.7	Ethylene-responsive transcription factor CRF2-like [Jatropha curcas]
scaffold0359_512732	5.1	9.3	Ethylene-responsive transcription factor ERF109-like [Jatropha curcas]
scaffold0447_369296	−2.2	−1.4	Ethylene-responsive transcription factor ERF023 [Jatropha curcas]
scaffold0636_609282	−1.4	1.3	Ethylene-responsive transcription factor ERF113 [Ricinus communis]
scaffold0668_410096	3.2	2.2	Ethylene-responsive transcription factor 5-like [Jatropha curcas]
scaffold0668_421116	5.3	3.2	Ethylene-responsive transcription factor 5-like [Jatropha curcas]
scaffold0770_505198	4.8	3.0	Ethylene-responsive transcription factor 5-like [Jatropha curcas]
scaffold0770_519202	2.6	2.0	Ethylene-responsive transcription factor 5-like [Jatropha curcas]
scaffold0782_27868	1.9	4.4	Ethylene-responsive transcription factor ERF061 [Jatropha curcas]
scaffold0838_409025	−1.3	−1.1	Ethylene receptor 2 [Ricinus communis]
scaffold1195_120325	2.5	−1.5	Ethylene-responsive transcription factor ERF017 [Jatropha curcas]
scaffold1267_104008	1.1	1.1	Ethylene-responsive transcription factor RAP2-7 isoform X3 [Ricinus communis]
scaffold2594_1826	−7.0	1.6	Ethylene-responsive transcription factor ERF010-like [Jatropha curcas]
**ABSCISIC ACID**			
scaffold0153_318575	−2.7	−1.3	Abscisic acid 8′-hydroxylase 2 [Ricinus communis]
scaffold0430_516715	−1.1	−1.1	Abscisic acid-insensitive 5-like protein 2 isoform X1 [Ricinus communis]
scaffold0748_467762	−1.3	−1.3	Abscisic acid receptor PYR1 [Ricinus communis]
scaffold2344_2623	−1.0	−1.6	Abscisic acid receptor PYL2 [Ricinus communis]
**GIBBERELLIN**			
scaffold0017_768934	−2.4	1.1	Gibberellin-regulated protein 14 isoform X3 [Jatropha curcas]
scaffold0194_369390	−1.3	−1.1	Gibberellin 20 oxidase 1-B like [Ricinus communis]
scaffold0291_1331456	1.3	1.3	Gibberellin 20 oxidase 1 [Ricinus communis]
scaffold0441_414170	0.6	2.2	Gibberellin 20 oxidase 2 like [Ricinus communis]
scaffold0801_379002	1.9	1.8	Gibberellin 20 oxidase 1 like [Populus trichocarpa]
scaffold0831_491806	1.6	−1.8	Feruloyl CoA ortho-hydroxylase 2 like [Populus euphratica]
scaffold1101_43630	3.9	2.1	Chitin-inducible gibberellin-responsive protein 1-like isoform X2 [Jatropha curcas]
scaffold1293_148625	2.5	1.2	Chitin-inducible gibberellin-responsive protein 1-like isoform X1 [Jatropha curcas]
scaffold2517_27680	−1.8	−2.1	Probable carboxylesterase 18 [Jatropha curcas]
**CYTOKININ**			
scaffold0045_49663	2.2	1.1	UDP-glycosyltransferase 76C3 like [Citrus clementina]
scaffold0199_1250120	−3.2	−1.3	Cytokinin hydroxylase like [Jatropha curcas]
**JASMONIC ACID**			
scaffold0015_736848	4.7	8.4	Protein TIFY 10A like JAZ2 [Hevea brasiliensis]
scaffold0026_2677018	0.1	1.0	Protein TIFY 3B like JAZ11 [Hevea brasiliensis]
scaffold0103_29681	−1.2	1.5	Transcription factor MYC4 like [Hevea brasiliensis]
scaffold0150_641	1.0	1.7	12-oxophytodienoate reductase 3 like [Hevea brasiliensis]
scaffold0762_419876	−0.9	1.4	Protein TIFY 3B like JAZ11 [Hevea brasiliensis]
scaffold0914_54940	1.2	1.2	Transcription factor bHLH35 isoform X2 [Jatropha curcas]
scaffold1038_209563	1.9	3.2	Allene oxide cyclase 3, chloroplastic-like [Populus euphratica]
scaffold1632_19091	−1.6	1.6	Latex allene oxide synthase [Hevea brasiliensis]

### Identification of transcription factors (TFs) in response to cold stress

Numerous families of transcription factors (TFs) are known to play crucial roles in signal transduction and regulation when plants are subjected with various abiotic stresses, including the AP2/EREBP, MYB, MYC, WRKY, NAC, and bHLH families (Agarwal and Jha, 2010; Liu et al., 2014). In the current study, 105 putative TFs were found to be exhibited differential expression in response to the cold treatment, of which those encoding AP2/EREBP domain-containing proteins constituted the largest group (22.8%), followed by MYB proteins (16.2%), WRKY proteins (8.6%), NAC proteins (5.7%), C2H2 proteins (4.7%), bHLHs (3.8%) (Table 2). The largest group of the cold-mediated TFs belonged to the AP2/EREBP family and was composed of 24 members. Of these, two genes (scaffold0997_153922, scaffold1276_47774) were annotated as CBF/ DREB genes, which have been shown to play important roles in cold acclimation leading to freezing tolerance (Nakashima et al., 2009). In addition, four members (scaffold0103_29681, scaffold0749_398268, scaffold0914_54940, scaffold1218_8594) of bHLH family were found to be exhibited differential expression in response to the cold treatment. Of those, the expression of an ICE1-like TF (scaffold1218_8594) was observed to up-regulated by cold treatment. Our results therefore indicated that the ICE-CBF pathway is conserved in rubber tree responses to the cold stress. Among the differentially expressed C2H2 TFs, all of the four members (scaffold0248_1439020, scaffold0540_449380, scaffold0760_285386, scaffold0827_2391, scaffold2824_5887) were up-regulated by cold treatment. We also noticed that the cold stress mediated the expression of TFs in the WRKY (scaffold0378_852023, scaffold0447_61208, scaffold0447_61719, scaffold0653_241031, scaffold0800_278767, scaffold0821_417450, scaffold0844_76279) family. The roles of members of these families in cold tolerance have been well established in numerous plants (Chinnusamy et al., 2010).

**Table 2:** Differential expression transcription factor (TF) in response to the cold treatment.

**Gene ID**	**Expression levels (log2ratio[cold/control])**		**Annotation**
	**Control-vs.-Cold 3 h**	**Control-vs.-Cold 12 h**	
**AP2-EREBP 24**			
scaffold0009_198930	1.4	−1.2	Ethylene-responsive transcription factor ERF034 like [Populus euphratica]
scaffold0024_3294824	1.5	−1.7	Ethylene-responsive transcription factor CRF2 like [Jatropha curcas]
scaffold0093_1433865	1.7	1.4	Ethylene-responsive transcription factor 4 like [Hevea brasiliensis]
scaffold0319_822155	4.0	3.1	Ethylene-responsive transcription factor 9 like [Hevea brasiliensis]
scaffold0342_1158497	2.3	1.6	Ethylene-responsive transcription factor 4 like [Hevea brasiliensis]
scaffold0359_512732	5.1	9.3	Ethylene-responsive transcription factor ERF109 like [Jatropha curcas]
scaffold0426_862663	4.2	1.8	Ethylene-responsive transcription factor ERF105 like [Hevea brasiliensis]
scaffold0426_899695	2.8	1.9	Ethylene-responsive transcription factor 5 like [Hevea brasiliensis]
scaffold0426_906054	1.6	1.5	Ethylene-responsive transcription factor 2 like [Jatropha curcas]
scaffold0447_369296	−2.2	−1.4	Ethylene-responsive transcription factor ERF023 like [Jatropha curcas]
scaffold0557_665510	1.3	1.6	Pathogenesis-related genes transcriptional activator PTI5 like [Ricinus communis]
scaffold0566_721951	1.2	2.4	Ethylene-responsive transcription factor RAP2-3 like [Hevea brasiliensis]
scaffold0636_609282	−1.4	1.3	Ethylene-responsive transcription factor ERF113 like [Ricinus communis]
scaffold0668_410096	3.2	2.2	Ethylene-responsive transcription factor 6 like [Jatropha curcas]
scaffold0668_421116	5.3	3.2	Ethylene-responsive transcription factor 6 like [Jatropha curcas]
scaffold0668_444732	2.6	3.6	Ethylene-responsive transcription factor 1A like[Hevea brasiliensis]
scaffold0770_505198	4.8	3.0	Ethylene-responsive transcription factor 6 like [Jatropha curcas]
scaffold0770_519202	2.6	2.0	Ethylene-responsive transcription factor 6 like [Jatropha curcas]
scaffold0782_27868	1.9	4.4	Ethylene-responsive transcription factor ERF061 like [Jatropha curcas]
scaffold0997_153922	5.0	6.7	Dehydration-responsive element-binding protein 1D like [Hevea brasiliensis]
scaffold1195_120325	2.5	−1.5	Ethylene-responsive transcription factor ERF017 like [Jatropha curcas]
scaffold1267_104008	1.1	1.1	Ethylene-responsive transcription factor RAP2-7 like [Ricinus communis]
scaffold1276_47774	2.2	2.6	Dehydration-responsive element-binding protein 2C like [Ricinus communis]
scaffold2594_1826	−7.0	−7.0	Ethylene-responsive transcription factor ERF010 like [Jatropha curcas]
**BES1**			
scaffold1038_185360	7.0	7.1	Beta-amylase 7 like protein BZR1 homolog 4 [Ricinus communis]
**bHLH**			
scaffold0103_29681	−1.2	1.5	Transcription factor MYC4 like[Hevea brasiliensis]
scaffold0749_398268	1.1	1.1	Transcription factor bHLH130 like transcription factor bHLH130-like [Jatropha curcas]
scaffold0914_54940	1.2	1.2	Transcription factor bHLH35 like [Jatropha curcas]
scaffold1218_85940	1.7	1.1	Transcription factor ICE1 like [Jatropha curcas]
**BSD**			
scaffold0565_438757	−1.1	−1.7	Uncharacterized protein LOC105630818 [Jatropha curcas]
**C2C2-CO-like**			
scaffold1446_82920	−1.1	−2.1	Zinc finger protein CONSTANS-LIKE 16 like [Ricinus communis]
**C2C2-GATA**			
scaffold0801_483822	−3.7	−3.6	GATA transcription factor 9 like [Jatropha curcas]
**C2H2**			
scaffold0248_1439020	3.6	3.1	Zinc finger protein ZAT10 like [Jatropha curcas]
scaffold0540_449380	5.2	4.8	Zinc finger protein ZAT10 like [Jatropha curcas]
scaffold0760_285386	2.4	4.0	Zinc finger protein ZAT12 like [Jatropha curcas]
scaffold0827_2391	3.2	2.6	Zinc finger protein ZAT10 like [Vitis vinifera]
scaffold2824_5887	2.3	1.7	Zinc finger protein ZAT10 like [Jatropha curcas]
**C3H**			
scaffold1776_58662	3.2	1.4	Zinc finger CCCH domain-containing protein 29 like [Jatropha curcas]
scaffold2028_37706	−1.6	−2.8	Splicing factor U2af small subunit A like[Jatropha curcas]
**CAMTA**			
scaffold0824_240435	1.1	−1.2	Calmodulin-binding transcription activator 4 like [Jatropha curcas]
**CPP**			
scaffold0649_61663	−1.5	−1.1	Protein tesmin/TSO1-like CXC 5 like [Jatropha curcas]
**E2F-DP**			
scaffold0190_833611	1.2	1.7	E2F transcription factor-like E2FE like [Ricinus communis]
**G2-LIKE**			
scaffold0064_1337771	1.5	1.2	Transcription factor BOA like uncharacterized protein LOC105641987 [Jatropha curcas]
scaffold0745_414068	−1.2	−1.2	Myb family transcription factor APL like [Jatropha curcas]
**GRAS**			
scaffold0781_525657	2.9	1.6	Scarecrow-like protein 33 like [Jatropha curcas]
scaffold1101_43630	3.9	2.1	Chitin-inducible gibberellin-responsive protein 1 like [Jatropha curcas]
scaffold1293_148625	2.5	1.2	Chitin-inducible gibberellin-responsive protein 1 like [Jatropha curcas]
scaffold3316_7246	−1.0	−2.2	Scarecrow-like protein 4 like scarecrow-like protein 4 [Jatropha curcas]
**HSF**			
scaffold0887_230318	−1.2	−1.6	Heat stress transcription factor B-3 like [Jatropha curcas]
**LIM**			
scaffold0024_1661357	−1.6	−1.1	Protein DA1 like protein DA1 isoform X1 [Jatropha curcas]
scaffold0430_259601	1.1	−1.7	Pollen-specific protein SF3 like [Jatropha curcas]
scaffold1673_9405	−2.5	−1.3	Pollen-specific protein SF3 like LIM domain-containing protein WLIM [Ricinus communis]
**LOB**			
scaffold0387_385506	−1.2	−1.4	LOB domain-containing protein 15 like [Ricinus communis]
scaffold0540_479077	−1.7	1.5	LOB domain-containing protein 38 like [Jatropha curcas]
scaffold0814_192853	−1.4	−2.7	LOB domain-containing protein 41 like [Jatropha curcas]
**MADS**			
scaffold0048_1495608	−11.8	−11.8	Developmental protein SEPALLATA 2 like [Jatropha curcas]
scaffold0824_196540	−1.1	−3.2	Agamous-like MADS-box protein AGL31 like[Betula platyphylla]
scaffold1181_25280	−1.6	−1.6	Agamous-like MADS-box protein AGL80 like [Jatropha curcas]
**mTERF**			
scaffold0077_598827	3.4	3.1	Uncharacterized protein LOC105641042 [Jatropha curcas]
scaffold0128_170927	−1.1	−1.3	Uncharacterized protein LOC8276547 isoform X1 [Ricinus communis]
scaffold0475_493935	−2.1	−2.3	Uncharacterized protein LOC105646130 [Jatropha curcas]
scaffold0794_215338	1.9	1.8	Conserved hypothetical protein[Ricinus communis]
**MYB**			
scaffold0037_481747	−1.7	−1.5	Myb-related protein 306 like [Jatropha curcas]
scaffold0064_1337771	1.5	1.2	Transcription factor BOA like [Jatropha curcas]
scaffold0167_1341204	6.8	3.4	Myb-related protein Myb4 like [Populus trichocarpa]
scaffold0214_973719	3.4	1.6	Protein RADIALIS-like 3 like [Populus trichocarpa]
scaffold0269_286149	1.1	1.1	Transcription factor MYB44 like[Jatropha curcas]
scaffold0393_760883	−1.4	−1.2	Myb-related protein 306 like [Ricinus communis]
scaffold0561_805801	−1.6	−1.0	Transcription factor MYB44 like [Jatropha curcas]
scaffold0745_414068	−1.2	−1.2	Myb family transcription factor APL like [Jatropha curcas]
scaffold0753_56980	−1.8	−1.1	Protein ODORANT1 like [Jatropha curcas]
scaffold0778_340533	2.0	1.4	Transcription factor RADIALIS like [Jatropha curcas]
scaffold0802_328595	1.8	2.6	Transcription factor TRY like [Populus euphratica]
scaffold0823_390156	5.6	5.2	Transcription factor RADIALIS like [Ricinus communis]
scaffold0926_151816	4.2	3.4	Transcription factor MYB44 like [Ricinus communis]
scaffold1291_85884	2.0	2.2	Transcription factor MYB44 like [Jatropha curcas]
scaffold1503_36079	1.2	1.3	Transcription factor MYB44 like[Jatropha curcas]
scaffold1604_48495	−2.0	−2.3	Protein ODORANT1 like protein ODORANT1 [Ricinus communis]
scaffold1881_19273	1.4	1.0	Transcription factor TT2 like [Eucalyptus grandis]
**NAC**			
scaffold0026_552629	−2.2	−1.0	NAC domain-containing protein 4 like [Jatropha curcas]
scaffold0026_564790	1.5	1.3	Protein FEZ like NAC domain-containing protein 89-like [Jatropha curcas]
scaffold0035_3053255	1.0	−1.1	NAC domain-containing protein 21/22 like [Manihot esculenta]
scaffold0807_44819	−2.6	−1.6	NAC transcription factor ONAC010 like [Ricinus communis]
scaffold0920_383574	3.0	2.2	NAC domain-containing protein 21/22 like [Manihot esculenta]
scaffold0926_71752	1.3	1.9	NAC domain-containing protein 100 like [Manihot esculenta]
**SBP**			
scaffold0896_63610	−1.3	−1.2	Squamosa promoter-binding-like protein 14 like [Jatropha curcas]
**SRS**			
scaffold1019_30037	−1.7	−3.1	Protein LATERAL ROOT PRIMORDIUM 1 like [Jatropha curcas]
**TIG**			
scaffold0824_240435	1.1	−1.2	Calmodulin-binding transcription activator 4 like [Jatropha curcas]
**Trihelix**			
scaffold0020_47233	−4.3	−3.5	Stress response protein NST1 [Jatropha curcas]
scaffold0442_841604	7.8	7.9	Trihelix transcription factor ASIL2-like [Jatropha curcas]
scaffold0626_598319	−1.9	1.5	Trihelix transcription factor ASIL1 [Jatropha curcas]
scaffold0753_500194	−1.8	−1.7	Stress response protein NST1 [Jatropha curcas]
**VARL**			
scaffold0194_575669	−1.0	−1.8	Histone-lysine N-methyltransferase ATX4 like [Jatropha curcas]
**WRKY**			
scaffold0189_35635	−4.9	−1.4	Probable WRKY transcription factor 11 like [Ricinus communis]
scaffold0378_852023	1.1	1.2	Probable WRKY transcription factor 70 like [Jatropha curcas]
scaffold0447_61208	4.2	2.0	Probable WRKY transcription factor 46 like [Populus trichocarpa]
scaffold0447_61719	4.2	2.0	Probable WRKY transcription factor 30 like [Populus trichocarpa]
scaffold0653_241031	3.7	2.3	Probable WRKY transcription factor 70 like [Jatropha curcas]
scaffold0800_278767	1.6	1.8	Probable WRKY transcription factor 27 like [Jatropha curcas]
scaffold0821_417450	3.4	1.2	Probable WRKY transcription factor 41 like [Populus trichocarpa]
scaffold0844_76279	3.3	1.1	Probable WRKY transcription factor 40 like [Populus trichocarpa]
scaffold3570_2572	−1.5	−1.9	Probable WRKY transcription factor 49 like [Jatropha curcas]
**zf-HD**			
scaffold0375_556816	2.0	2.3	Zinc-finger homeodomain protein 4 like [Populus euphratica]
scaffold0682_603876	1.5	1.7	Zinc-finger homeodomain protein 4 like [Populus euphratica]
scaffold0853_204606	−2.3	−1.1	Zinc-finger homeodomain protein 11 like [Ricinus communis]
scaffold1106_14241	1.4	1.7	Zinc-finger homeodomain protein 5 like [Ricinus communis]

## Discussion

Cold is the major environmental abiotic stress adversely affecting the growth and geographical distribution of plants. *Hevea brasiliensis* is native to tropical rainforests in the Amazonian basin, but was expanded to sub-optimal environments worldwide in the late 1970s, prominently northeast India, southern China, highland and coastal areas of Vietnam, and southern Brazil. Cold stress has therefore become a limiting factor not only of rubber production, but also survival of rubber trees. Chilling temperatures (0–15°C) interfere with a number of metabolic and physiological processes such as chloroplast and mitochondria integrity, plastid membrane composition and photosynthetic electron transport, resulting in leaf wilt and lesions, bark breakdown and latex leakage, root system withering and frostbite, and ultimately cell death. Increased cold tolerance is therefore a major aim of rubber tree breeding programs.

As an upstream regulator, ICE1 plays a key role in cold signaling pathways in a wide range of plants species such as *Arabidopsis* (Shi Y. et al., 2012; Lang and Zhu, 2015), wheat, rice, *Phalaenopsis aphrodite, Trifoliate orange*, banana and grapevine (Badawi et al., 2008; Nakamura et al., 2011; Peng et al., 2013, 2014; Xu et al., 2014; Huang X. S. et al., 2015). It is therefore reasonable to expect that ICE1 homologs also play a key role in cold tolerance in *H. brasiliensis*. Characterization and functional analysis of the *ICE1* gene in rubber trees is therefore a significant step in determining its cold signaling pathway.

Multiple sequence alignments indicate that HbICE1 shares typical ICE1 protein domains with other plant species such as the MYC-like basic helix-loop-helix (bHLH) domain, serine-rich region (S-rich), zipper region (ZIP) and ACT-UUT-ACR-like domain; however, it possesses a varied N terminus (Figure 1). A potential sumoylation site, which is reportedly crucial for AtICE1 activation and stability (Miura et al., 2007), was also found in HbICE1 (Figure 1), suggesting that HbICE1 activity is mediated by the SUMO E3 ligase. Furthermore, HbICE1 was confirmed as being nuclear localized, and able to bind to the MYC-recognition element. These observations imply that HbICE1 is a novel putative ICE1 homolog.

Expression profiles revealed that, like *AtICE1, HbICE1* is expressed constitutively in all tissues (Chinnusamy et al., 2003). Furthermore, *HbICE1* was induced by multiple abiotic stresses including cold, dehydration, wound and salinity (Figure 5). *HbICE1* was only slightly induced by cold, consistent with a previous report suggesting that cold stress induces little transcriptional alteration, instead resulting in posttranslational modification of ICE1 to active the CBF pathway (Chinnusamy et al., 2003; Ding et al., 2015). Upregulation of *HbICE1* following dehydration was consistent with previous reports in *P. trifoliate* and *Pyrus ussuriensis* (Huang X. et al., 2015; Huang X. S. et al., 2015), but differs from *AtICE1*, which is not triggered by dehydration (Chinnusamy et al., 2003). This disparity between *HbICE1* and *AtICE1* might be attributed to the inherent differences between plant species.

Cold acclimation is one of the major mechanisms for plant to adapt to cold stress (Thomashow, 1999). Indeed, cold acclimation was found to enhance freezing tolerance rubber tree after exposure to low temperature (Figure S2).The electrolyte leakage was less in the cold-acclimated (CA) rubber trees to the nonacclimated (NA) rubber tree, and CA rubber trees experienced less cold stress-induced H_2_O_2_ accumulation compared to the NA plants, suggesting an enhanced membrane integrity and the lower cold stress-induced H_2_O_2_ accumulation in the CA rubber tree seedlings to NA plants. Overexpression of *HbICE1* in *Arabidopsis* enhanced cold tolerance only after cold acclimation (Figure 6), indicating that other co-factors associated with cold acclimation are essential for *HbICE1*-mediated cold tolerance. Similar results were reported for wheat ICE genes (Badawi et al., 2008), where overexpression of *TaICE87* or *TaICE41* in *Arabidopsis* enhanced freezing tolerance only after cold acclimation (Badawi et al., 2008). These results suggest that other co-factors induced by cold acclimation are essential for HbICE1-mediated cold tolerance. Previous reports also confirmed this hypothesis. Chinnusamy et al. (2003) showed that cold-induced modification of the AtICE1 protein or a transcriptional cofactor is necessary for AtICE1-induced activation of CBF expression. Furthermore, Miura et al. (2007) showed that SIZ1 (SAP and Miz1), mediates sumoylation of ICE1, which reduces the polyubiquitination of ICE1 to enhance its stability. A potential sumoylation site was also found in HbICE1 protein (Figure 1), suggesting that HbICE1 activity could be regulated by sumoylation via the SUMO E3 ligase.

Physiological parameters such as electrolyte leakage and contents of MDA, chlorophyll and proline are closely related to cold tolerance under the regulation of *COR*, which is triggered by the ICE transcription factor (Badawi et al., 2008; Peng et al., 2014; Xu et al., 2014; Liu et al., 2017). In many plant species, overexpression of *ICE1* is sufficient to alter physiological parameters and enhance cold tolerance (Feng et al., 2013; Huang X. et al., 2015). In our study, the *HbICE1ox* transgenic lines showed improved survival rates and freezing tolerance as a result reduced electrolyte leakage and MDA metabolism, and increased proline accumulation (Figure 7), suggesting that *HbICE1* plays a positive regulatory role in the response to cold stress. These findings suggest that the *ICE-CBF-COR* transcriptional cascade, which influences the freezing tolerance capacity of plants, exists not only in *Arabidopsis* but other species such as *H. brasiliensis*.

H_2_O_2_ is often associated with biotic and abiotic stresses (Yuan and Huang, 2016; Cao et al., 2017; Lu et al., 2017; Yuan et al., 2017). Our study further suggests that the *35S::HbICE1* plants experienced less cold stress-induced H_2_O_2_ accumulation compared to the WT, despite similar contents under normal conditions. Consistence with the decrease in MDA content and electrolyte leakage under cold stress, these data imply that enhanced membrane integrity and decreased levels of lipid peroxidation caused the lower cold stress-induced H_2_O_2_ accumulation in the transgenic compared to WT plants.

Transcription factors families such as *AP2/EREBP, MYB, WRKY, C2H2, NAC*, and *bHLH* are well known to be involved in stress tolerance in plants (Chinnusamy et al., 2010), but how they work synergistically to cope with cold tolerance requires determination. In this work, two genes (scaffold0997_153922, scaffold1276_47774) annotated as CBF/DREB and one ICE1 member of bHLH family (scaffold1218_8594) were up-regulated by cold treatment (Table 2), indicating that ICE-CBF pathway is conserved in rubber tree responses to the cold stress. The phytohormones auxin, ABA, JA, and ethylene are known to play important roles in the regulation of plant growth and abiotic stress responses (Shi Y. et al., 2015). In current study, the DGEs data suggested that many phytohormones related genes were responsive to cold treatment in rubber tree. ABA integrates various stress signals and modulates stress responses, but whether it is involved in cold responses is still debated (Tuteja, 2007). It has been suggested that plant's response to the cold stress may be ABA-independent, but increasing evidences of contrary were reported (Lang et al., 1994; Wang et al., 2015). We noticed that ABA 8′-hydroxylase, a key enzyme in ABA catabolism, was down-regulated by cold treatment. Down-regulation of the ABA 8′-hydroxylase gene implies that catabolism of ABA might be attenuated in rubber tree under cold stress, which is consistent with the previous report that ABA levels increase slightly in response to low temperature (Lang et al., 1994). In addition, we detected the decreased expression of ABA receptor *PYR1* and *PYL2* (Table 1), how ABA mediated signaling is involved in the cold responses of rubber tree remains further investigation. Ethylene has been well documented in the cold stress response (Shi Y. et al., 2012, 2015). In our study, the expression of *ERFs* was significantly regulated by cold treatment. JA was recently reported to significantly enhance plant freezing tolerance with or without cold acclimation, (Hu et al., 2013). We observed the increased expression of several key genes for JA biosynthesis, including *OPR* (scaffold0150_641), *AOS* (scaffold1038_209563) and *AOC* (scaffold1632_19091), and several essential genes (e.g., *JAZs* and *MYC*) in JA signaling pathways at 12 h after cold treatment, strongly suggesting that cold stress triggers JA biosynthesis and responses in rubber tree, which is consistent with the study of *A. thaliana* (Hu et al., 2013). JA was therefore proposed to be a positive regulator of cold responses in rubber tree.

In conclusion, a novel ICE-like transcription factor, designated HbICE1, was isolated from *H. brasiliensis* and functionally characterized. This nuclei protein, which has typical features of ICE proteins, was found to have transactivation activity via binding to MYC recognition sites. *35S::HbICE1* plants showed enhanced cold resistance via increased proline content, a reduction in MDA metabolism and electrolyte leakage, and a decrease in ROS accumulation, promoting expression of the cold-responsive genes. These findings suggest that *HbICE1* is a member of the *ICE* gene family and positive regulator of cold tolerance. Differentially expressed genes (DEGs) analysis showed that cold treatment changed genes expression profiles involved in many biological processes and phytohormones perception and transduction. Ethylene, JA, ABA, as well as ICE-CBF signaling pathways might work synergistically to cope with cold tolerance in rubber tree. These findings will help elucidate the cold signaling network in *H. brasiliensis*, ultimately aiding breeding programs aimed at improving cold stress tolerance.

## Author contributions

HY conceived and directed this study, designed and performed the experiments, analyzed the data, wrote and revised the manuscript; YS, YL, MO, XT, and WC performed the experiments, analyzed the data; XH revised the manuscript. All authors have read and approved the final version submitted.

### Conflict of interest statement

The authors declare that the research was conducted in the absence of any commercial or financial relationships that could be construed as a potential conflict of interest.

## Supplementary material

Figure S1 GO functional classification on DEGs in pairwise of Control-vs.-Cold 3 h **(A)** and Control-vs.-Cold12 h **(B)**. X axis means number of DEGs (the number is presented by its square root value). Y axis represents GO terms. All GO terms are grouped in to three ontologies: blue is for biological process, brown is for cellular component and orange is for molecular function.

Figure S2 Electrolyte leakage **(A)** and DAB staining **(B)** of the rubber tree seedlings with indicated freezing temperatures. Seedlings were treated at −16°C for 0.5 h or 1 h for nonacclimated (NA) and cold-acclimated (CA) plants (CA; 1 day at 4°C). Error bars show SD from three replicates. (^**^*P* < 0.01, ^***^*P* < 0.005, student's *t*-test).

Table S1 List of the primers used for qRT-PCR.

Table S2 Summary of sequencing data for each sample.

Table S3 Alignment statistics of reads align to reference genome.

Table S4 Differentially Expressed Genes (using Noiseq) in pairwise of Control-VS-Cold 3 h.

Table S5 Differentially Expressed Genes (using Noiseq) in pairwise of Control-VS-Cold 12 h.

## Abbreviations

GFP: green fluorescent protein
*ICE1*: Inducer of CBF Expression 1
MDA: malondialdehyde
qRT-PCR: quantitative real-time PCR
ROS: reactive oxygen species.
