# Supplementary figures and images for: Overexpression of Hevea brasiliensis HbICE1 Enhances Cold Tolerance in Arabidopsis
Source: Front Plant Sci. 2017 Aug 22;8:1462. doi: 10.3389/fpls.2017.01462 (PMC5572258; doi:10.3389/fpls.2017.01462)

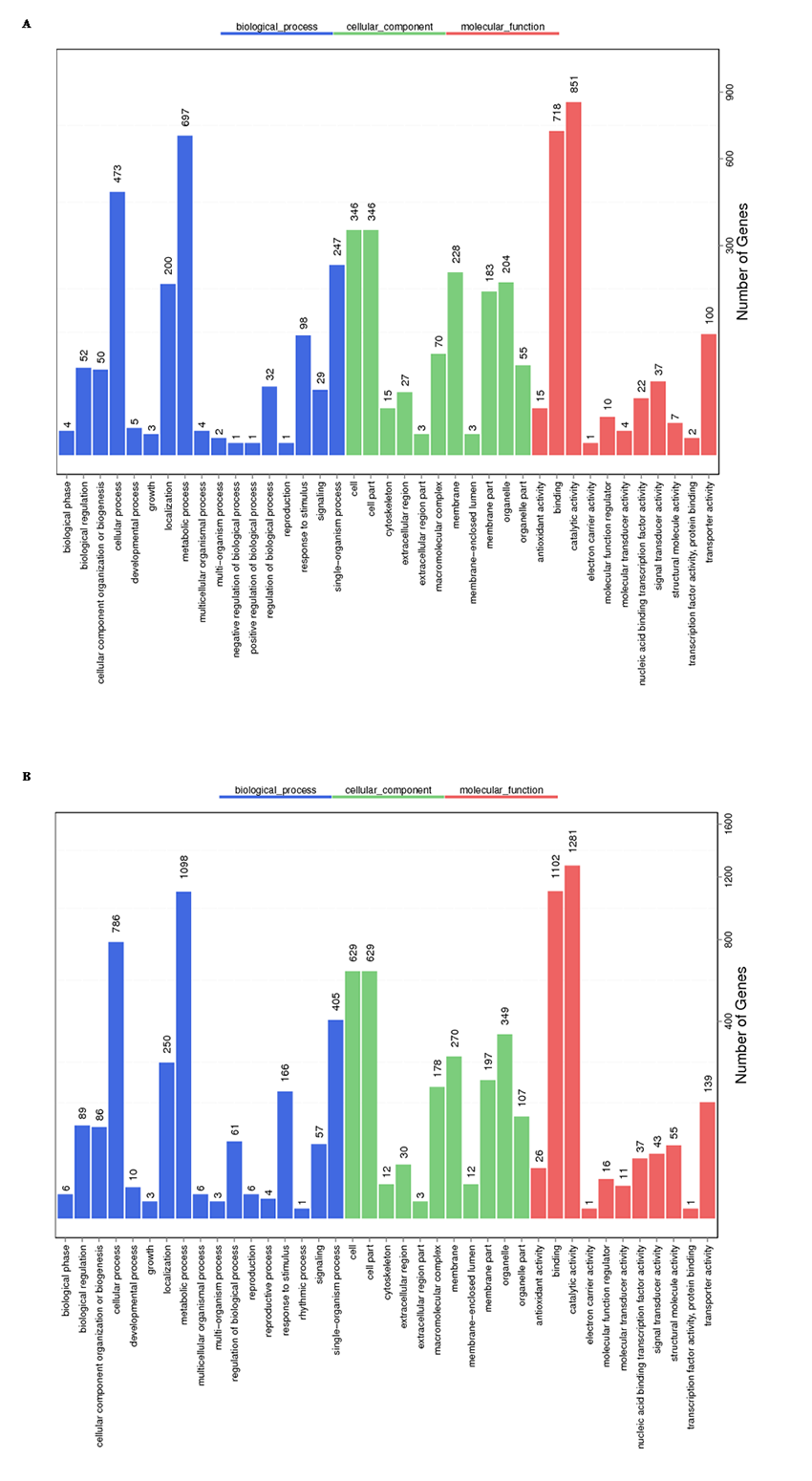

Supplement: Figure S1 — GO functional classification on DEGs in pairwise of Control-vs.-Cold 3 h (A) and Control-vs.-Cold12 h (B). X axis means number of DEGs (the number is presented by its square root value). Y axis represents GO terms. All GO terms are grouped in to three ontologies: blue is for biological process, brown is for cellular component and orange is for molecular function. [file Image1.TIF]

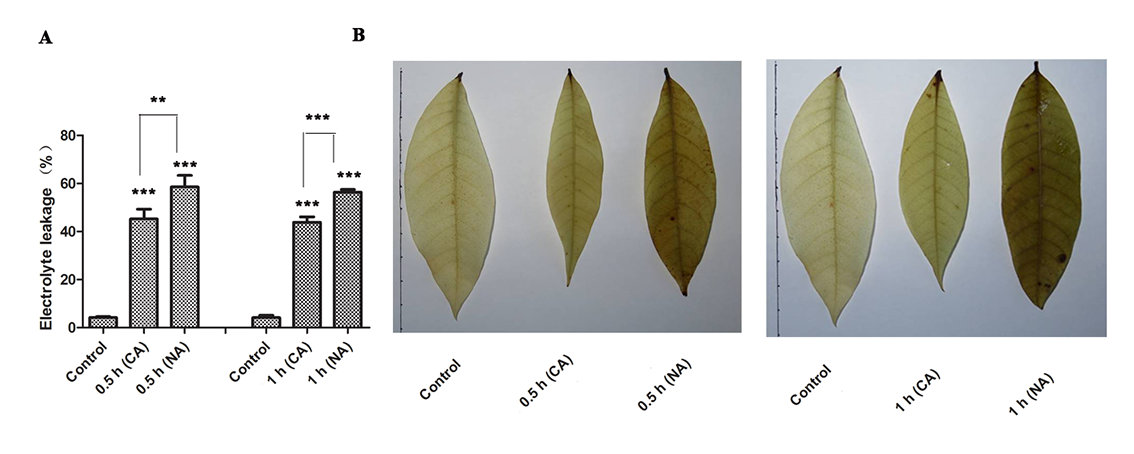

Supplement: Figure S2 — Electrolyte leakage (A) and DAB staining (B) of the rubber tree seedlings with indicated freezing temperatures. Seedlings were treated at −16°C for 0.5 h or 1 h for nonacclimated (NA) and cold-acclimated (CA) plants (CA; 1 day at 4°C). Error bars show SD from three replicates. (**P < 0.01, ***P < 0.005, student's t-test). [file Image2.TIF]
