# Supplementary material for: Overexpression of Hevea brasiliensis HbICE1 Enhances Cold Tolerance in Arabidopsis
Source: Front Plant Sci. 2017 Aug 22;8:1462. doi: 10.3389/fpls.2017.01462 (PMC5572258; doi:10.3389/fpls.2017.01462)
Supplement: Table S1 — List of the primers used for qRT-PCR. [file Table1.DOCX]

**Table. S1** List of the primers used for qRT-PCR.

**Primer Sequence (5’ to 3’)**

HbICE1 qRT-F TTTTAGTGGGCCCGAGTTC

HbICE1 qRT-R ATTGCGGCAGCATTATCG

AtCOR15AqRT-F GGCGTATGTGGAGGAGAAAG

AtCOR15AqRT-R CCCTACTTTGTGGCATCCTTAG

AtCOR47qRT-F GGCTGAGGAGTACAAGAACAA

AtCOR47qRT-R ACAATCCACGATCCGTAACC

AtKIN1qRT-F GCAATGTTCTGCTGGACAAG

AtKIN1qRT-R TCCTTCACGAAGTTAACACCTC

AtRD29AqRT-F GCTTTCTGGAACAGAGGATGTA

AtRD29AqRT-R CGACTCTTCCTCCAACGTTATC

AtPDF2qRT-F TAACGTGGCCAAAATGATGC

AtPDF2qRT-R GTTCTCCACAACCGCTTGGT

AtEIF4qRT-F GCACAGTTTGATGATGCACGTCAGT

AtEIF4qRT-R GGTTCTCTTGAAGACCCATGGCA
