# Supplementary material for: Overexpression of Hevea brasiliensis HbICE1 Enhances Cold Tolerance in Arabidopsis
Source: Front Plant Sci. 2017 Aug 22;8:1462. doi: 10.3389/fpls.2017.01462 (PMC5572258; doi:10.3389/fpls.2017.01462)
Supplement: Table S2 — Summary of sequencing data for each sample. [file Table2.DOCX]

| Sample | Sequencing Strategy | Raw Data Size (bp) | Raw Reads Number | Clean Data Size (bp) | CleanReads Number | Clean Data Rate (%) |
| --- | --- | --- | --- | --- | --- | --- |
| Cold 12 h_1 | SE50 | 1206671400 | 24133428 | 1205039300 | 24100786 | 99.86 |
| Cold 12 h_2 | SE50 | 1201107350 | 24022147 | 1194809400 | 23896188 | 99.47 |
| Cold 12 h_3 | SE50 | 1201008500 | 24020170 | 1199808950 | 23996179 | 99.9 |
| Cold 3 h_1 | SE50 | 1206585600 | 24131712 | 1201318650 | 24026373 | 99.56 |
| Cold 3 h_2 | SE50 | 1206499800 | 24129996 | 1202862000 | 24057240 | 99.69 |
| Cold 3 h_3 | SE50 | 1206857850 | 24137157 | 1204912250 | 24098245 | 99.83 |
| Control_1 | SE50 | 1206625750 | 24132515 | 1205524550 | 24110491 | 99.9 |
| Control_2 | SE50 | 1206561400 | 24131228 | 1204377750 | 24087555 | 99.81 |
| Control_3 | SE50 | 1206873300 | 24137466 | 1204215200 | 24084304 | 99.77 |
